# Transcriptomic and Proteomic Analyses Reveal New Insights into Regulatory Mechanisms of Strontium in Bovine Chondrocytes

**DOI:** 10.3390/ani13081301

**Published:** 2023-04-11

**Authors:** Fangyuan Zeng, Lan Li, Jiaqi Yang, Siqi Liu, Yang Yuan, Chenxu Zhao, Jianguo Wang

**Affiliations:** College of Veterinary Medicine, Northwest A&F University, Xianyang 712100, China

**Keywords:** strontium, bovine chondrocyte, transcriptomic, proteomics

## Abstract

**Simple Summary:**

Strontium (Sr) is one of the essential microelements of the body, which mainly exists in the bone. Sr has been demonstrated to affect the formation of bone by regulating the development of cattle chondrocytes, which make cartilage. However, the molecular mechanism by which Sr regulates the growth of bovine chondrocytes is unknown. In this study, we investigated 111 differentially expressed genes and 286 differentially expressed proteins. Sr was involved in pathways related to proliferation and differentiation of chondrocytes, fat metabolism, the inflammation process, and immune responses of chondrocytes. This study described the potential regulatory mechanism of Sr in bovine chondrocytes and provided new insights into the functions and application of Sr in ruminants.

**Abstract:**

Strontium (Sr) is a trace element found mainly in bone, and it performs a dual action by promoting bone formation and inhibiting bone resorption. Sr has been used to evaluate the gastrointestinal calcium (Ca) absorption capacity of dairy cows due to the similar physicochemical properties of the two elements. However, the possible effects of Sr on dairy cows remain unclear. This study aimed to explore the potential regulatory mechanism of Sr in bovine chondrocytes by performing transcriptomic and proteomic analyses. A total of 111 genes (52 up-regulated and 59 down-regulated) were identified as significantly altered (1.2-fold change and *p* < 0.05) between control and Sr-treated groups. Moreover, LC-MS-based proteomic analysis detected 286 changed proteins (159 up-regulated and 127 down-regulated) between the control and Sr-treated groups (1.2-fold change and *p* < 0.05). Gene Ontology (GO) and Kyoto Encyclopedia of Genes and Genomes (KEGG) annotations of a combination analysis of the transcriptomic and proteomic data revealed that the genes were predominantly involved in chondrocyte proliferation and differentiation, fat metabolism, the inflammation process, and immune responses. Overall, our data reveal a potential regulatory mechanism of strontium in bovine chondrocytes, thus providing further insights into the functions and application of Sr in ruminants.

## 1. Introduction

Sr is an alkaline earth metal belonging to the IIA group of elements. Sr primarily exists as salt compounds in nature, such as strontium sulfate (SrSO_4_) and strontium carbonate (SrCO_3_) [1]. It can also occur as divalent cations, which can bind to proteins present in bodily fluids [2]. Several previous biological studies on Sr mainly focused on bones since Sr, like Ca, is a bone-seeking element. Sr is mainly stored in the bones and teeth after absorption by cells and then contributes to the process of bone formation [3]. Sr plays a significant role in promoting osteoblast-mediated bone formation and inhibiting osteoclast-mediated bone resorption [4,5,6]. Sr has attracted significant attention in recent years due to the development of the antiosteoporosis drug, strontium ranelate (Sr RAN) [7]. It has been demonstrated that Sr can effectively increase bone mass and strength by causing the thickening of the cortical bone and an increase in the number of trabeculae [8]. Therefore, Sr can be very effective in the prevention and treatment of vertebral fractures impaired by osteoporosis [9], and Sr-doped bone repair material has exhibited a good performance in repairing bone defects caused by bone tumors or injuries [10]. In addition, in a veterinary clinic, Hyde et al. reported that Sr concentration could serve as an index for the Ca absorption capacity of the gastrointestinal tract in ruminants, with a close correlation found between the absorption rates of oral Sr and radioactive Ca in both cows and sheep [11,12]. Hence, we aimed to determine the potential effects of Sr on other tissues in the body.

The dynamic balance of bone formation and absorption ultimately determines the osteogenesis and development of bone. A number of recent studies have demonstrated that many, perhaps the majority, of bone cells are derived from chondrocytes via direct transformation [13]. Chondrocytes can effectively differentiate from chondroprogenitor cells, which originate from bone-marrow mesenchymal stem cells (BMSCs) and can develop into hypertrophic chondrocytes. Thereafter, hypertrophic chondrocytes can be gradually replaced by osteoblasts when the cartilage matrix is partially calcified [14]. The different cellular characteristics and expression profiles of chondrocytes are altered during chondrocyte maturation and characteristic molecules are expressed to regulate bone formation in a complex process. According to the limited studies involving rodents, Sr also plays a role in cartilage and chondrocytes during bone formation. These studies suggest that Sr can weaken cartilage degeneration and cause subchondral bone remodeling in osteoarthritis. The cartilage matrix quality was also improved through combined treatment with Sr RNA and mechanical vibration in rats [15,16]. Sr RAN has been shown to accelerate cartilage regeneration by inhibiting the Wnt/β-catenin signaling pathway [17] and to stimulate human chondrocytes to synthesize components of the cartilage matrix and alleviate cartilage degradation in osteoarthritis [18]. In addition, several studies have also established that Sr is involved in regulating chondrocyte maturation [19,20,21]. However, research on the effect of Sr in the bones of ruminants is relatively rare.

Transcriptomic and proteomic techniques serve as valuable tools for exploring key expression changes to achieve a more complete understanding of the molecular mechanisms underlying Sr-treated bovine chondrocytes. The objective of our study was to investigate the potential molecular mechanisms related to Sr in bovine chondrocytes. Hence, we analyzed and compared the transcriptomic and proteomic profiles of control and Sr-treated groups of bovine chondrocytes.

## 2. Materials and Methods

The Northwest A&F University Institutional Animal Care and Use Committee approved all procedures involving animals in this study. A commercial dairy cow farm located in Shaanxi Province, China, was selected as the location of this study.

### 2.1. Animals and Tissue Collection

In this study, newborn Holstein calves (*n* = 3, 1 day old, male, 38.0 ± 2.8 kg BW, fasting) were used. The calves were euthanized with barbiturates by the resident veterinarian. The patellar cartilage of the knee joint was surgically removed, placed in pre-cooling PBS, and transferred to the laboratory for subsequent cell separation within 15 min after euthanasia.

### 2.2. Isolation and Culture of Bovine Primary Chondrocytes

Primary chondrocytes were isolated from the patellar cartilage of each of the three calves and cultured separately. The cartilage surface of the patella was exposed after removing the excess surrounding tissue and was then cut into thin slices. Thereafter, the cartilage slices were incubated with 0.25% (*m/v*) type II collagenase (catalogue no. 17101015, Gibco, CA, USA) at 37 °C in 5% CO_2_ for 18 h. The cell suspension was filtered with a 100-mesh filter to remove the incompletely digested tissue, and then centrifuged at 400× *g* for 10 min. The obtained chondrocytes were inoculated in 60 mm culture dishes (catalogue no. 704001, Nest, Wuxi, China) at a concentration of 5 × 10^5^ cells/mL. The culture medium used was DMEM/high sugar (catalogue no. 12800017, Gibco, CA, USA) with 10% fetal bovine serum (FB15015, Clark, VA, USA) and was replaced regularly every 2 d. The cells were then digested with 0.25% (*m/v*) trypsin digestive solution (catalogue no. T1186, Gentihold, Beijing, China) and subcultured until the density of the cells reached 80~90% (Figure S1). The formal test was carried out with the second-generation cells with high purity and viability. The maximum blood Sr concentration is approximately 1 μg/mL in dairy cows [12]. In our previous study, 1 μg/mL was set as the medium-dose group. Doses of 0.1 μg/mL and 10 μg/mL were used for the low- and high-dose groups. Sr was previously determined to promote the proliferation and inhibit the differentiation of bovine chondrocytes in a dose-dependent manner via the growth factor β/SMAD signaling pathway [22]. For the present experiments, cells of the Sr group were treated with 10 μg/mL of Sr (SrCl_2_·6H_2_O, dissolved in 0.9% (*m/v*) NaCl solution, catalogue no. V900279, Sigma, MO, USA) for 4 h and those of the control group were treated with vehicle (0.9% (*m/v*) NaCl solution). Finally, the cells were collected in a 1.5 mL tube and the number of cells in each tube was more than 1 × 10^7^. The tubes were immediately frozen by placing them into liquid nitrogen, and then were stored at −80 °C until further processing. Cell samples collected from the three newborn Holstein calves were processed separately for proteomics and transcriptomics.

### 2.3. RNA Extraction, Sequencing, and Analysis

Total RNA from the bovine chondrocytes was extracted using a Trizol reagent kit (catalogue no. 15596018, Invitrogen, CA, USA), and the extraction procedure was performed according to the manufacturer’s instructions. The extracted total RNA was assessed using an Agilent Bioanalyzer 2100 (Agilent, CA, USA); the RIN number of all samples was higher than 9 and met the requirements for subsequent trials (Table S1, Figure S2). The sequencing libraries were thereafter constructed and sequenced using an Illumina HiseqTM 2500/4000 by Gene Denovo Biotechnology Co., Ltd. (Guangzhou, China). After removing the low-quality reads and adapter sequences, the various clean reads were aligned to the bovine reference genome sequences UMD3.1 (ftp://ftp.ensembl.org/pub/release-104/fasta/bos_taurus/dna/ (accessed on 12 September 2021)). The various genes obtained were quantified using the FPKM (Fragments Per Kilobase of transcript per Million mapped reads) method. The transcripts between the control group and the Sr-treated group with the parameter of the false discovery rate (FDR) < 0.05 and absolute fold change (FC) ≥ 1.2 were considered differentially expressed genes (DEGs). The row sequencing data generated from this study were deposited in NCBI SRA (http://www.ncbi.nlm.nih.gov/sra accessed on 6 January 2023) under the BioProject ID PRJNA877584. The identified DEGs were subsequently subjected to enrichment analysis of GO with Goseq software and KEGG with KOBAS 2.0 software. The various significantly enriched GO terms and KEGG pathways were defined with a hypergeometric test (corrected *p*-value < 0.05).

### 2.4. Protein Extraction, Sequencing, and Analysis

The total protein of the bovine chondrocytes was extracted using lysis buffer (2% SDS, 8 mol/L urea, 1 mg/mL protease inhibitor cocktail). After vortexing and lysis for 30 min on ice and centrifugation at 1400× *g* for 15 min at 4 °C, the supernatant was collected. The protein concentration of the supernatant was quantified using a BCA (catalogue no. P0010, Beyotime, Shanghai, China) protein assay kit. Sequence-grade modified trypsin (Promega, WI, USA) was used to digest 100 μg proteins to peptides, which were then labeled using an iTRAQ-8Plex Isobaric Mass Tag Labeling Kit (Thermo Fisher Scientific, Waltham, MA, USA) following the manufacturer’s instructions. The labeled peptide mixture was subjected to high-pH reverse-phase separation, and then the separated peptide fractions were analyzed using low-pH nano-HPLC-MS/MS (Orbitrap Fusion) in data-dependent acquisition mode (DDA).

The clean data from the mass spectra were transformed into Mascot Generic Format (MGF) files using Proteome Discovery 1.2 (Thermo, Pittsburgh, PA, USA) and analyzed using the Mascot search engine (Matrix Science, London, UK; version 2.3.2). The peptide threshold for protein identification was 1.0% FDR. The differentially expressed proteins (DEPs) were analyzed via a *t*-test (1.2-fold change and *p* < 0.05). In addition, DEPs were annotated against GO and KEGG databases to analyze the various functions of the proteins.

### 2.5. Association Analysis of Transcriptomic and Proteomic Data

Association analysis was performed for the data mined between the transcriptomic and proteomic data of the bovine chondrocytes. A nine-quadrant map and GO/KEGG association analysis were used to assess the potential correlation between the gene expression levels in the transcriptome and corresponding proteins in the proteome. The results were then divided into three distinct categories: DEGs and DEPs had the same expression trend, DEGs and DEPs displayed a reverse expression trend, and DEGs and DEPs showed no difference in expression. Transcriptomic and proteomic GO/KEGG association analysis was used to analyze GO function and KEGG pathway information in the transcriptomic and proteomic data to compare the similarities and differences between the two groups of gene functions and metabolic pathways.

## 3. Results

### 3.1. RNA-Seq Transcriptomic and Quantitative Proteomic Analyses

A total of 9727 transcripts were integrated from the clean data, and 111 genes (among which 52 were up-regulated and 59 were down-regulated) were identified as significantly altered (1.2-fold change and *p* < 0.05) between the control and Sr-treated groups. The LC-MS-based proteomic analysis detected 286 differentially altered proteins (among which 159 were up-regulated and 127 were down-regulated) between the control and Sr-treated groups (1.2-fold change and *p* < 0.05). The summaries of all differentially expressed genes and proteins are presented in Tables S2 and S3, respectively.

### 3.2. Gene Ontology Analysis of the Differentially Expressed Transcripts and Proteins

**Figure 1:**
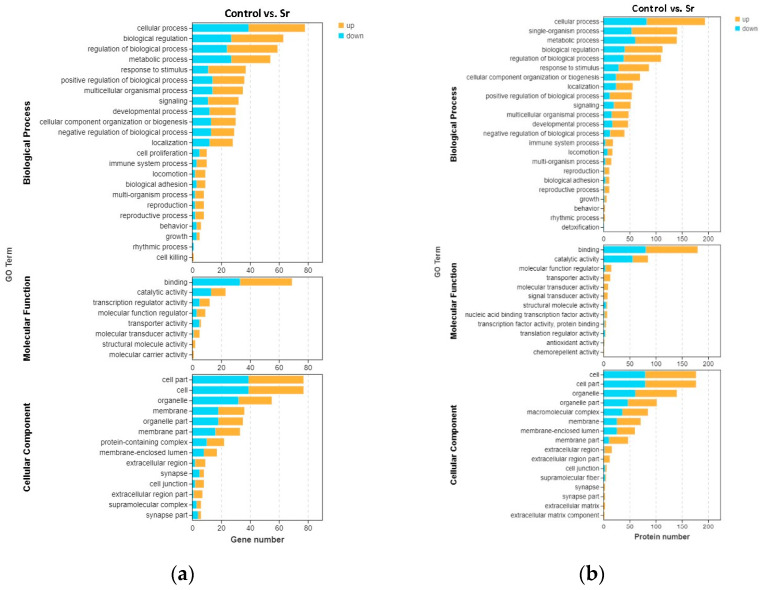
GO categories of the differentially regulated transcripts (**a**) and proteins (**b**) in bovine chondrocytes between the control and Sr-treated groups. The ordinate is the GO terms, and the abscissa indicates the number of the differentially expressed genes annotated to GO terms. The blue bars represent down-regulated molecules, whereas the yellow bars represent up-regulated molecules.

The GO annotation analysis of the mRNA and protein assigned to each of the three main GO terms and secondary categories is presented in Figure 1. A total of 111 differentially regulated transcripts were annotated with 45 distinct GO terms: 51.11% belonged to biological processes, 17.78% belonged to molecular functions, and the remaining 31.11% belonged to cellular components. The differentially expressed proteins were assigned 51 distinct GO terms. Among these GO terms, molecular functions accounted for 23.53% of the differentially regulated proteins, cellular components accounted for 31.37%, and biological processes reflected the vast majority of the up-regulated and down-regulated proteins (45.10%). The most frequently encountered biological processes in the transcriptomic and proteomic data were cellular process (70.27% and 71.85%), biological regulation (56.76% and 41.85%), and the regulation of biological process (53.15% and 40.74%). The most abundant function was that of binding (62.16% and 66.67%) at both mRNA and protein levels. Cell parts (69.37% and 65.56%) and cells (69.37% and 65.56%) were dominant in the cellular component category for both mRNA and protein. The ratio of GO terms of the transcriptome was relatively similar to that of the proteome. This similarity is important because, although the gene and protein alterations did not necessarily agree with the analysis data, the conclusions reached from the functional studies between RNA- and protein-based evaluations demonstrated minimal actual differences.

### 3.3. Function Correlation Analysis Using the KEGG System

**Figure 2:**
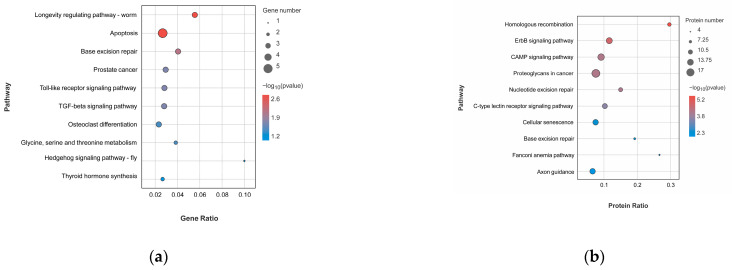
KEGG-enriched pathways of the differentially regulated transcripts (**a**) and proteins (**b**) in bovine chondrocytes between the control and Sr-treated groups. Each circle represents a pathway. The ordinate is the name of the pathway and the abscissa serves as the enrichment factor. The color of the circle represents the q-value, which is the p-value after the multiple hypothesis test correction. The circle size indicates the number of target genes enriched in the pathway. Pathways with redder colors and larger circles exhibited greater reference values. Ten pathways with minimum q-values were displayed in this study.

In the KEGG pathway analysis, the various differentially regulated transcripts were assigned to 136 different pathways (Figure 2a). Most classifications included longevity regulating pathway worm, apoptosis, base excision repair, prostate cancer, toll-like receptor signaling pathway, and TGF-β signaling pathway.

However, in the proteome data, differentially regulated proteins were annotated to 198 different pathways (Figure 2b). The KEGG analysis showed that the differentially regulated proteins were mainly associated with homologous recombination, ErbB signaling pathway, cAMP signaling pathway, and proteoglycans in cancer.

### 3.4. Association Analysis of the Transcriptomic and Proteomic Data

**Figure 3:**
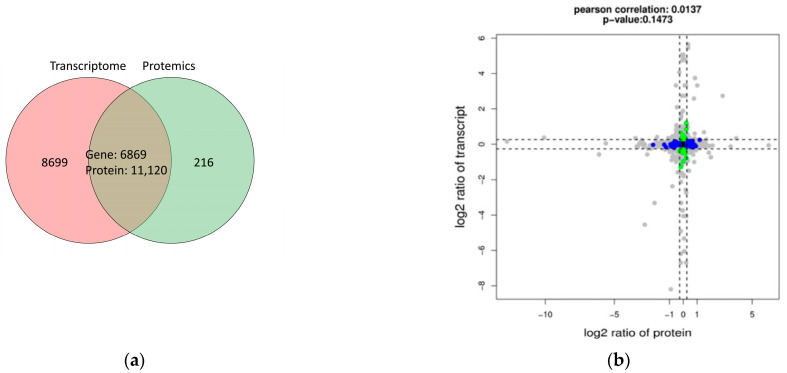
Combination analyses involving the transcriptomic and proteomic data identified differential expression of the various genes and proteins. (**a**) Venn diagram of the combined analyses of transcriptomic and proteomic data; the overlap indicates the commonly expressed molecules at the mRNA and protein levels. (**b**) Nine-quadrant map of combination analyses of the transcriptomic and proteomic; the abscissa is the fold change (Fc) of the protein (log 2) and the ordinate is the Fc of protein (log 2). The top panel of the map shows the Pearson correlation and *p*-value. Each point represents a specific gene or a protein: a black point indicates non-differentially expressed proteins and genes, a green point means differentially expressed in the gene but not in protein, a blue point indicates expressed in protein but not in the gene, a gray point means that the condition of Fc > 1.2, *p* > 0.05 is met.

The comparison between the transcriptomic and proteomic data identified 6869 expressed genes and 11120 expressed proteins in common at both the mRNA and protein levels (Figure 3a). As the 9-quadrant map in Figure 3b shows, 43 non-differentially expressed proteins (NDEPs) conjoined with DEGs, 245 non-differentially expressed genes (NDEGs) conjoined with DEPs, and no significantly expressed genes were found in common between the transcriptomic and proteomic data.

**Figure 4:**
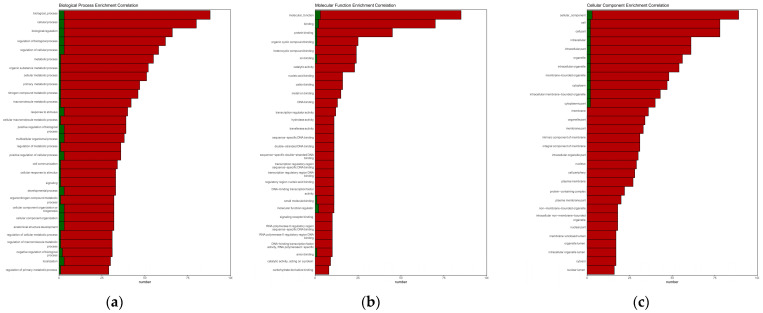
GO categories of the combined analysis of the transcriptomic and proteomic data. The differentially expressed molecules were classified into biological process (**a**), molecular function (**b**), and cellular component (**c**). The ordinate serves as the GO terms, and the abscissa indicates the number of the differentially expressed genes annotated to GO terms. The green bars represent differentially expressed genes, whereas the red bars represent differentially expressed proteins.

We performed GO classification for transcriptomic and proteomic data (Figure 4). The biological process enrichment correlation showed that the differentially expressed molecule was mainly classified as a cellular process, biological regulation, regulation of the biological process, and regulation of the cellular process. The molecular function enrichment correlation showed that the terms binding, protein binding, and organic cycle compound binding were classified as a molecule, which was differentially expressed to the greatest extent. The cellular component enrichment correlation indicated that the differentially expressed molecule was mainly classified as a cell, cell part, intracellular, etc. Although a very low correlation was found between the transcriptomic and proteomic data, the pathways involved in both the DEGs and DEPs were identified using KEGG pathway analysis (Table 1). The pathways included those of fat digestion and absorption, vitamin digestion and absorption, peroxisome proliferator-activated receptor (PPAR) signaling pathway, and cholesterol metabolism.

## 4. Discussion

Sr can exhibit the dual action of promoting bone formation and inhibiting bone resorption [23] and has been widely used in treating osteoporosis in the form of Sr RAN. Our previous findings show that Sr can significantly promote the proliferation and inhibit the differentiation of bovine chondrocytes by modulating the TGF-β/SMAD signaling pathway [22]. The current study was designed to better characterize the response of bovine chondrocytes upon exposure to Sr by performing transcriptomic and proteomic analysis of chondrocytes cultured with Sr.

A total of 111 transcripts and 286 proteins were found to be differentially expressed in the bovine chondrocytes between the control and Sr-treated group. The combination analysis showed a relatively low concordance (r = 0.0137) with no differentially expressed gene in common between the transcriptomic and proteomic data in this study. A series of prior studies have shown that a lack of correspondence between RNA and proteins in the transcriptomic and proteomic association analysis is a general problem in cases where the analyses are primarily based on RNA transcriptomic and proteomic data [24,25]. It could be related to the complex post-transcriptional control and translation regulation [26]. It might be plausible to explain how Sr can regulate bovine chondrocytes if the conducted research had been only mRNA-based or protein-based. These results indicated that more studies evaluating the potential overlap between the chondrocyte’s protein and mRNA dynamics are needed to evaluate the reliability of mRNA-based analyses of bovine chondrocytes. Given that the analysis of the GO terms was linked with substantial overlap, thereby suggesting that the conclusions at the functional level were similar even though the correlation of differential expression of mRNA and proteins was relatively poor, the objective of our study was to understand the KEGG pathways screened using the combined analysis of the transcriptomic and proteomic data. 

The results appeared to support the function of Sr in regulating the proliferation and differentiation of chondrocytes. Three DEGs, including inhibitor of nuclear factor kappa-B kinase subunit alpha (IKBKA), fos-like antigen 1 (FOSL1), and lymphocyte cytosolic protein 2 (LCP2), were classified in osteoclast differentiation. FOSL1, a major component of the dimeric transcription factor activator protein-1 (Ap-1), is considered an essential enzyme for the cellular development of bone formation and maintenance [27]. FOSL1 can be regulated by the osteoclast differentiation factor receptor activator of nuclear factor-κ B ligand (RANKL), OPG ligand (OPGL), and tumor necrosis factor (TNF)-related activation-induced cytokine (TRANCE) [28]. In our study, the downregulation of Ap-1 was related to FOSL1, suggesting that, as expected, Sr can play a key role in inhibiting osteoclast differentiation. In addition, three down-regulated transcripts were observed to be related to the TGF-β signaling pathway, which is another major pathway involved in the metabolism, differentiation, proliferation, and survival of chondrocytes [29]. SMAD1/5/9 can also play a significant role by regulating a series of biological processes in the TGF-β pathway with the changes in their phosphorylation levels [30]. Thus, decreased levels of SMAD1/5/9 transcript suggest reduced osteoclast differentiation under the regulation of Sr. In addition, a previous study in our laboratory showed that Sr can dose-dependently reduce SMAD1/5/9 phosphorylation in bovine chondrocytes, which indicated that the TGF-β pathway is important for Sr to exert its diverse biological functions. Moreover, two DEGs and one DEP were classified as belonging to the phosphatidylinositol 3’-kinase (PI3K)-Akt signaling pathway, which is responsible for regulating various physiological functions [31]. The PI3K-Akt signaling pathway is involved in modulating different biological functions, such as chondrocyte proliferation, cartilage protection, and cartilage degeneration, and the hypothesis that Sr is involved in the regulation of chondrocytes is further supported [32]. The results collectively suggested a reduced chondrocyte differentiation of the Sr-treated group that supports the positive effect of Sr in the biological function of bone.

The increased protein abundance of apolipoprotein A (APOA)-1 was involved in fat digestion and absorption, vitamin digestion and absorption, and the PPAR signaling pathway. This observation appears to be logical given the inhibitory effects of Sr in effectively reducing adipocyte differentiation in multipotent mesenchymal cells (MMCs) [33]. Osteoporosis has been reported to promote adipogenesis in bone marrow [34], and the reduction in the accumulation of marrow fat is a mechanism that can exert bone-protective effects [35]. Thus, we can speculate that the antiosteoporosis action of Sr might be related to the regulation of fat metabolism. However, further research is needed to confirm this finding and better understand how Sr can potentially participate in the regulation of marrow fat.

The results show that some of the DEGs and DEPs between the control group and Sr-treated group were mapped in a wide range of pathways such as those associated with apoptosis, toll-like receptor signaling pathway, complement and coagulation cascades, and Th1 and Th2 cell differentiation, thereby suggesting that Sr might be involved in the regulation of the inflammation process and immune responses. The anti-inflammatory function of Sr in cartilage has been suggested in a study related to Sr-doped biomaterials [36], and Sr RNA has been revealed as a potential treatment option for osteoarthritis [37]. However, the mechanisms related to the potential involvement of Sr in the inflammation process and immune responses are still not clear due to limited studies, and further research is needed to understand and validate the biological roles of Sr.

## 5. Conclusions

The results of transcriptomic and proteomic analyses determined that Sr is involved in three crucial biological processes in bovine chondrocytes. Sr has a positive effect on the proliferation and differentiation of chondrocytes via the TGF-β signaling pathway, SMAD1/5/9 pathway, and PI3K-Akt signaling pathway. The regulatory effect of Sr on fat metabolism genes further suggests that the antiosteoporosis action of Sr might be related to the inhibition of fat accumulation. Sr was also shown to be involved in apoptosis, the toll-like receptor signaling pathway, complement and coagulation cascades, and Th1 and Th2 cell differentiation, which may be related to the regulation of inflammation processes and immune responses. Overall, this study of the regulatory mechanisms of strontium in bovine chondrocytes provides more theoretical support for the safety of using Sr to assess Ca absorption and its potential application in dietary supplements.

## Figures and Tables

**Table 1:** KEGG pathway enrichment analysis in combination with the transcriptomic and proteomic data.

Pathway ID	Pathway	Candidate Genes with Pathway Annotation	Candidate Proteins with Pathway Annotation	Gene Value	Pro-Value
ko04975	Fat digestion and absorption	0	1	1.000	0.028
ko04977	Vitamin digestion and absorption	0	1	1.000	0.028
ko05143	African trypanosomiasis	1	1	0.686	0.029
ko03320	PPAR signaling pathway	0	1	1.000	0.038
ko04979	Cholesterol metabolism	0	1	1.000	0.038
ko04512	ECM–receptor interaction	1	1	0.686	0.038
ko04610	Complement and coagulation cascades	2	1	0.686	0.038
ko04510	Focal adhesion	1	1	0.717	0.094
ko05205	Proteoglycans in cancer	0	1	1.000	0.094
ko05165	Human papillomavirus infection	2	1	0.712	0.097
ko04151	PI3K-Akt signaling pathway	2	1	0.717	0.097
ko04210	Apoptosis	5	0	0.188	1.000
ko04212	Longevity regulating pathway worm	3	0	0.188	1.000
ko03410	Base excision repair	3	0	0.302	1.000
ko05215	Prostate cancer	3	0	0.410	1.000
ko04350	TGF-β signaling pathway	3	0	0.410	1.000
ko04620	Toll-like receptor signaling pathway	3	0	0.410	1.000
ko04380	Osteoclast differentiation	3	0	0.508	1.000
ko00260	Glycine, serine, and threonine metabolism	2	0	0.508	1.000
ko04658	Th1 and Th2 cell differentiation	2	0	0.686	1.000

## Data Availability

The row sequencing data generated from this study have been deposited in NCBI SRA (http://www.ncbi.nlm.nih.gov/sra accessed on 25 September 2022) under the BioProject ID PRJNA877584.

## Supplementary Materials

The following supporting information can be downloaded at: https://www.mdpi.com/article/10.3390/ani13081301/s1, Table S1: Overall results for RNA samples; Table S2: Differentially expressed genes; Table S3: Differentially expressed proteins; Figure S1: The morphology of bovine primary chondrocyte with varied culturing time; Figure S2: Results of total RNA quality inspection.
