# Supplementary material for: Transcriptomic and Proteomic Analyses Reveal New Insights into Regulatory Mechanisms of Strontium in Bovine Chondrocytes
Source: Animals (Basel). 2023 Apr 11;13(8):1301. doi: 10.3390/ani13081301 (PMC10135116; doi:10.3390/ani13081301)
Supplement: Supplementary file 1 [file animals-13-01301-s001.zip › Figures S1 and S2.pdf]

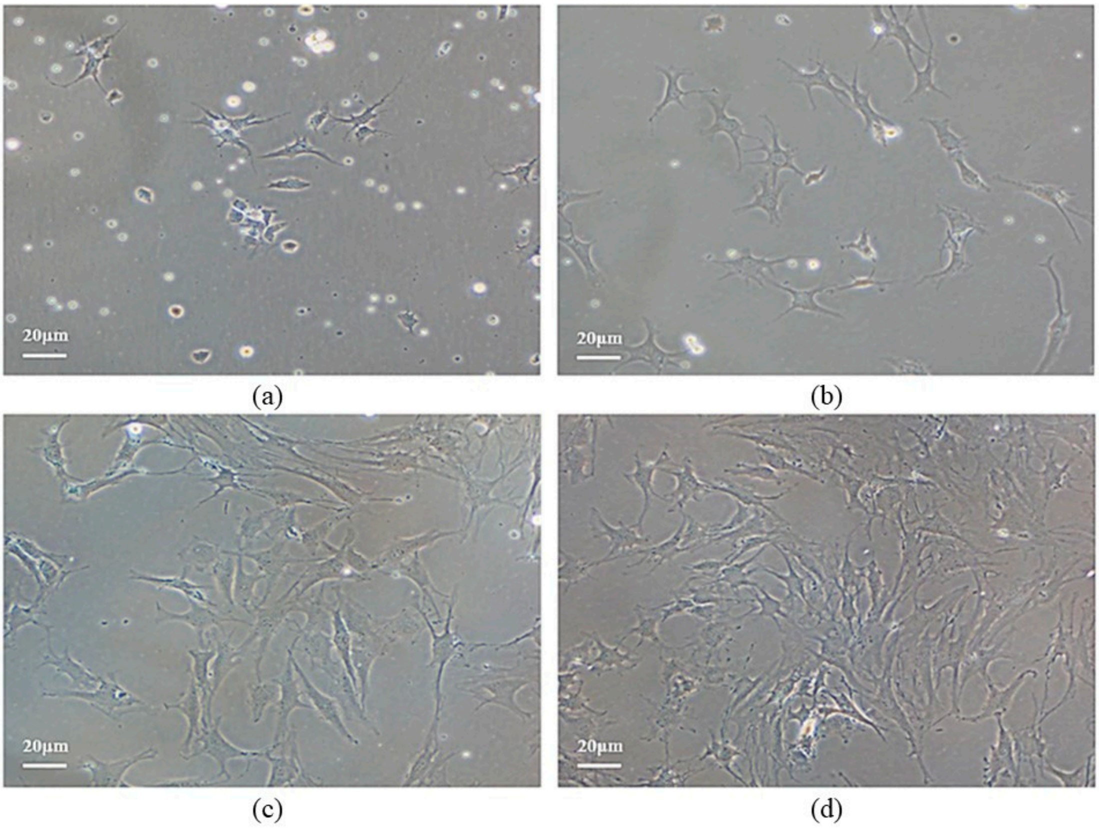

Figure S1: The morphology of bovine primary chondrocyte culturing with different time (200×). (a): 1 d; (b): 3 d; (c): 5 d; (d): 7 d.

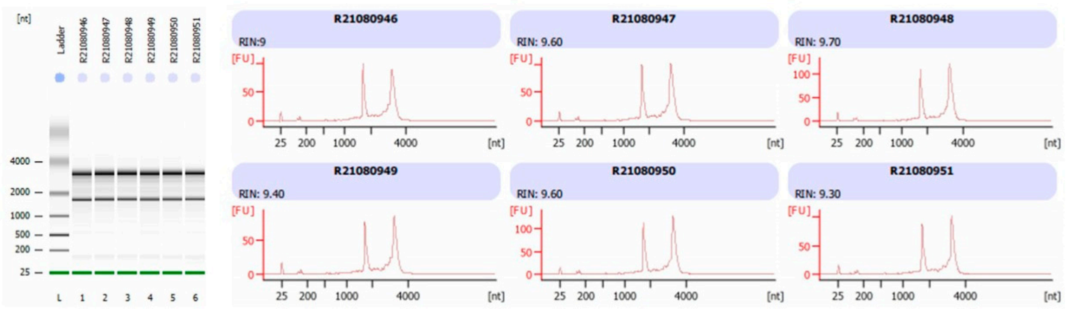

(a) (b)

Figure S2: The quality inspection of total RNA using Agilent Bioanalyzer 2100. (a): Gel electrophoresis; (b): electropherogram analysis.
